# Margin Matters: Advances in Intraoperative Margin Assessment for Breast-Conserving Surgery

**DOI:** 10.3390/diagnostics15212804

**Published:** 2025-11-05

**Authors:** Valentin Ivanov, Usman Khalid, Rosen Dimov

**Affiliations:** 1 Medical Simulation and Training Center, Medical Faculty, Medical University of Plovdiv, Department of Surgery, University Hospital “Kaspela”, 4001 Plovdiv, Bulgaria; 2 Medical Faculty, Medical University of Plovdiv, 4002 Plovdiv, Bulgaria; usmankhalid957@gmail.com; 3 Department of Special Surgery, Medical Faculty, Medical University of Plovdiv, 4001 Plovdiv, Bulgaria; rosendimov68@gmail.com

**Keywords:** breast cancer, breast-conserving surgery, intraoperative margin assessment, frozen section analysis, optical coherence tomography, artificial intelligence

## Abstract

**Background/Objectives**: Breast cancer is the most prevalent neoplasm in women. Improved screening and systemic therapies have allowed more patients to choose breast-conserving surgery over mastectomy. However, preserving glandular tissue while achieving negative margins remains difficult. Traditional intraoperative margin assessment techniques like frozen section analysis, cavity shave margins, intraoperative ultrasonography, and specimen radiography aim to reduce positive margins and re-excision rates but face several limitations, including time consumption, interpretive challenges, and operator dependency. Our aim was to critically evaluate both conventional and emerging intraoperative margin assessment techniques in breast-conserving surgery, highlighting their clinical utility, limitations, and potential to reduce re-excision rates and improve patient outcomes. **Methods**: We assessed PubMed and Google Scholar databases using search terms such as specimen radiography, intraoperative ultrasonography, mass spectrometry, optical coherence tomography, artificial intelligence, and others. Studies were selected based on relevance, language, and completeness, and refined through author consensus. **Conclusions**: Conventional techniques have demonstrated value in reducing re-excisions and preserving cosmetic outcomes. Emerging tools like MarginProbe, fluorescence imaging, mass spectrometry (MasSpec Pen, iKnife), OCT, and AI-enhanced imaging show promise in offering real-time feedback and higher diagnostic accuracy. However, high costs, training needs, and data variability limit their widespread adoption. Investment in standardised protocols and multicentre trials is essential. Integration of imaging, spectroscopy, and AI may offer the most robust framework for improving surgical outcomes and quality of life for breast cancer patients.

## 1. Introduction

When it comes to incidence and death, breast cancer is the most prevalent neoplasm in women. The improved accessibility of screening programs, which results in more frequent early diagnoses and new systemic medicines, is responsible for the growing trend in both incidence and survival. Patients’ quality of life is subsequently enhanced because of better results and the potential to choose conservative surgery over mastectomy [1].

The “no ink on the tumour” definition of a negative margin for invasive breast cancer undergoing lumpectomy is advised by the most recent guidelines from the American Society of Clinical Oncology, the Society of Surgical Oncology, the American Society for Radiation Oncology, and the National Comprehensive Cancer Network. A surgical excision of the primary tumour and a small portion of surrounding disease-free tissue with maximum tissue preservation and negative margins is known as breast conserving surgery [2].

Oncoplastic surgery aims to achieve a good aesthetic result preserving radicality, not necessarily only through tissue sparing. The main and difficult goal of oncoplastic surgery is still to preserve as much healthy glandular tissue as possible to improve aesthetic results while still attaining negative margins for oncological reasons. When tissue is not removed during lumpectomies to preserve glandular tissue, the free margin width between the tumour and healthy tissue is drastically reduced. This increases the number of positive margins and necessitates surgical re-excision [3].

For intraoperative diagnosis during breast cancer surgery (BCaS), the conventional options include intraoperative pathologic techniques, such as CSM and frozen section analysis. Additionally, intraoperative imaging has historically been limited to intraoperative ultrasonography and specimen radiography. Positive margin rates may be reduced by these techniques. However, several issues must be resolved before the method can be widely used, including the time-consuming and intricate nature of these pathologic procedures and the burden that pathologists are under. Researchers have been looking for new methods lately. Recently, numerous techniques for intraoperative real-time control of breast margins using live tissue have been developed. These techniques include optical coherence tomography, fluorescence imaging, mass spectrometry-based instruments (such the MasSpec Pen and iKnife), radiofrequency spectroscopy (MarginProbe device), and artificial intelligence-enhanced imaging [4].

Our aim was to critically evaluate both conventional and emerging intraoperative margin assessment techniques in breast-conserving surgery, highlighting their clinical utility, limitations, and potential to reduce re-excision rates and improve patient outcomes.

## 2. Materials and Methods

A comprehensive literature search was performed using PubMed (Medline) and Google Scholar to identify impactful publications on intraoperative margin assessment techniques in breast-conserving surgery. The search covered studies published between 4 May 2025, and 4 August 2025, including all earlier indexed literature available within these databases up to that period.

The review followed a structured narrative framework. The initial scoping phase (4 May–20 May 2025) was used to identify major thematic areas and refine keyword combinations related to intraoperative margin evaluation. The full electronic search (21 May–10 June 2025) was then conducted using the terms specimen radiography, intraoperative ultrasonography, mass spectrometry, optical coherence tomography, artificial intelligence, cavity shave margins, frozen section analysis, intraoperative margin assessment, and breast-conserving surgery.

All search results were imported into reference management software (EndNote 21), and duplicate entries were automatically and manually removed. Between 11 June and 5 July 2025, two reviewers independently screened titles and abstracts for relevance, followed by full-text eligibility assessment between 6 July and 25 July 2025. Any disagreements were resolved by discussion and consensus. The final synthesis and verification of included studies were completed by 4 August 2025, with manual cross-referencing of bibliographies to identify additional relevant publications.

Inclusion criteria were as follows:

1. Studies evaluating various intraoperative margin assessment techniques in patients undergoing breast-conserving surgery.
2. Full-text original articles or review articles written in English and containing the standard sections (introduction, materials and methods, results, and discussion with conclusions).
3. Studies included irrespective of tumour subtype, and both invasive breast carcinoma and ductal carcinoma in situ (DCIS) were eligible. Margin standards for invasive disease and DCIS were considered according to their respective guideline definitions during data extraction and synthesis.

Exclusion criteria were as follows:

1. Articles not available in full text, conference abstracts, preprints, and case reports.
2. Non-English publications.
3. Studies not reporting relevant data on margin assessment techniques or their impact on oncological outcomes.

**Figure 1:**
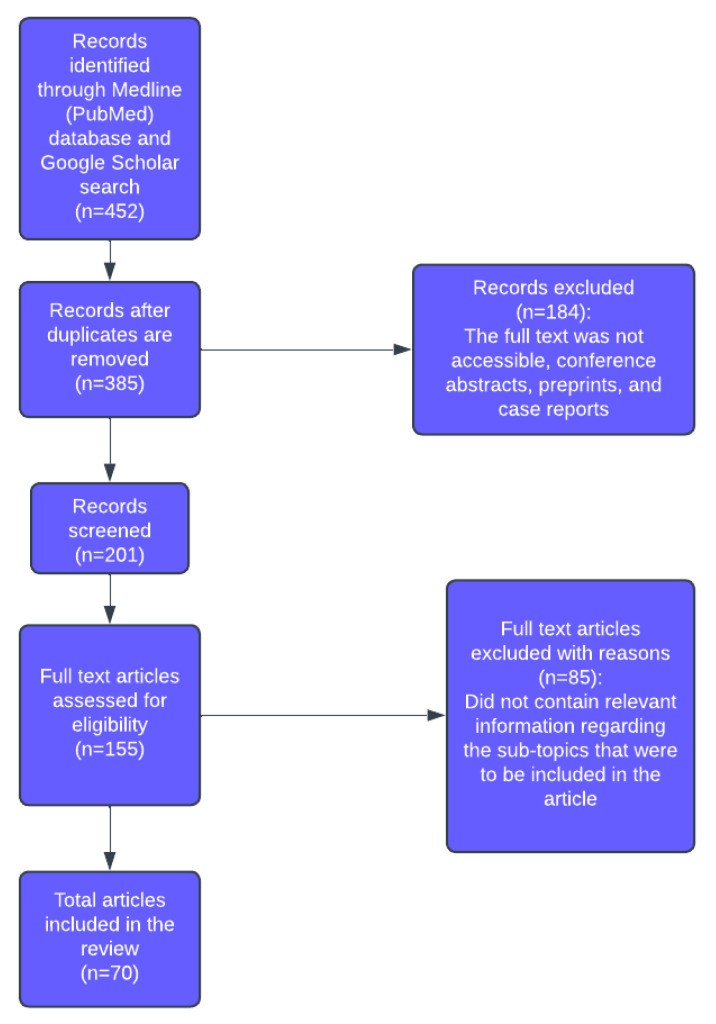
PRISMA-like flow diagram summarising the selection process of studies included in the review.

A total of 452 records were initially identified. After removal of duplicates, 385 records remained. Following title and abstract screening, 184 records were excluded. Of the 155 full-text articles assessed for eligibility, 85 were excluded for not meeting the inclusion criteria. A total of 70 articles were included in the final review. The article selection process is summarised in Figure 1.

## 3. Results

### 3.1. Conventional Pathology Based Techniques

#### 3.1.1. Frozen Section Analysis

Frozen section analysis (FSA) is no longer considered the standard intraoperative approach in many centres, and the definition of close margins is no longer regarded as critical in invasive breast cancers, with current guidelines adopting no ink on tumour’ standard. Frozen section procedures typically add a median duration of 20 min with the specimen being evaluated by a pathologist. The process involves a 1 cm marginal excision, which is histologically assessed and divided into positive, close, or negative. Patients with close or positive margins are indicated for re-excision, whilst two or more consecutive non-negative margins indicate a mastectomy. A randomised control trial (RCT) assessed 60 patients that underwent BCaS for in situ or invasive carcinoma with (FSA). Good concordance was displayed between results of FSA and the final paraffin section in assessing margin status with 20 patients achieving a primary negative margin with the remaining 40 subjects undergoing additional excisions at the time of initial surgery due to these margins. Of these 40 patients, in 32 patients a negative margin could be achieved with re-excisions. The great advantage of intra-operative evaluation of margin status is the immediate re-excision during the same operation, which prevents change in tissue morphology over a fixed period if re-excision is required in a separate surgery. Furthermore, by aiding in the measurements of clearance distance, surgeons have increasingly precise margins to differentiate healthy and tumorous tissue allowing for more informed intraoperative decisions and not merely relying on a simple binary result [5]. Lowering reoperation rates remains a key driving force in the utilisation of frozen section margin assessment, which in turn lowers patient anxiety and enhances quality of life. Furthermore, a rise in BCaS can lead to better aesthetic results and occasionally avoid mastectomy entirely. This prevents delays in the initiation of adjuvant therapy and saves money on extra procedures and hospital stays [6]. A meta-analysis study evaluated the accuracy of different intraoperative techniques for margin assessment reporting a sensitivity of 86% and a specificity of 91% with 97% of heterogeneity for the frozen section technique [7]. Furthermore, data from the National Surgical Quality Improvement Program at the Mayo clinic highlighted that a 70% relative risk reduction is possible when employing FSA (3.6% reoperation rate compared to a national rate of 13.2%) reinforcing the diagnostic accuracy of histopathological techniques [8]. Tissue preservation remains both a physiological and cosmetic priority, especially near the nipple and chest wall. Identifying specific margins allows for targeted re-excision as opposed to wide cavity shavings. A study conducted by Noguchi et al. evaluated the diagnostic value of intraoperative histologic examination of frozen sections of surgical margins and axillary lymph nodes (ALN) in 95 patients with breast cancer who underwent breast-conserving surgery. The periphery of the excised breast tissue was peeled like an orange and examined histologically by frozen section. Results highlighted a diagnostic accuracy of 87%, a sensitivity of 96%, and a specificity of 84% with the ALNs obtaining a diagnostic accuracy of 97%, a sensitivity of 77%, and a specificity of 100% with frozen section [9]. An analysis of 1102 patients undergoing BCT demonstrated that intraoperative FSA significantly reduced reoperation rates due to residual tumour, with conversion to mastectomy required in 5.9% of patients compared to 9.7% when frozen section was not utilised. Although its use increased operative time, it improved margin clearance and reduced the need for secondary procedures [10]. Similarly, a retrospective cohort study of 328 patients reported that intraoperative frozen section histology enabled one-step oncologically complete surgery in most cases, avoiding reoperation in 18% of patients and resulting in significant cost savings of approximately 11.7% per patient compared to a hypothetical scenario [11]. Despite its advantages, FSA has notable drawbacks. Sampling errors contribute to false negatives, particularly in sentinel lymph nodes or heterogeneous tissue [12]. Certain benign breast lesions can mimic carcinoma histologically, leading to interpretive errors and false positives if not carefully examined [13]. Differentiating atypical ductal hyperplasia from DCIS, lobular carcinoma, or post-NACT changes can be challenging, reducing reliability in these scenarios [14]. Freezing itself may introduce crush, cautery, or folding artefacts, especially in fatty tissue, degrading slide quality, and diagnostic clarity [15]. Moreover, FSA can prolong surgery by 20–50 min and requires resolute pathology staff and infrastructure, which limits cost-effectiveness in low baseline positive-margin settings [16].

#### 3.1.2. Cavity Shaving

Cavity shaving is best regarded as a surgical technique rather than a margin assessment tool. It is an independent pathology-based approach involving minimal resection of the breast lesion and extension of all margins to achieve total clearance of residual breast tissue. Although it was initially introduced to examine residual activity following mastectomy, studies highlighted its capability to decrease the positive margin rate and re-excision rate [3,17]. A single centre retrospective study assessed 594 patients subjected to cavity shave. There was a significant reduction in positive, focal positive, or closer margins reported at 8.9% vs. 18.5 with operation time being reduced in patients subjected to cavity shave. The multivariate analysis intraoperative evaluation of sentinel lymph node and cavity shave were both predictive factors quicker a quicker surgery. Decreased operation times contribute to a reduction in the costs, better resource utilisation, and shorter time of anaesthesia, therefore, less operative, and immunological stress [18]. Positive margins are key factors which contribute to the need for re-excision, therefore by tackling the issue at its roots, the overall number of secondary operations will be reduced. In a randomised study, patients who underwent cavity shaving had significantly lower rates of positive margins (19% vs. 34%) and re-excision (10% vs. 21%) compared to those who did not. In addition, there were no significant differences in complication rates between the groups. Cosmesis is also a major aspect to consider when implementing surgical techniques [19]. A separate study by Mook et al. compared tissue volume removed in cavity shave margins (CSM) compared to a standard mastectomy and found that patients in the CSM group had significantly less tissue removed compared to the standard partial mastectomy group (80.7 cm^3^ vs. 165.1 cm^3^). Fewer re-excisions were needed in the CSM group (18.1% vs. 34.6%), and cosmetic outcomes were enhanced with a more desirable average cosmetic score (2.3 vs. 3.0) [20]. A large multicentre RCT conducted across nine U.S. centres further reinforced these findings. Among 396 patients randomised intraoperatively, those in the cavity shave group demonstrated significantly lower rates of positive margins (9.7% vs. 36.0%) and re-excision or mastectomy (8.7% vs. 23.5%) compared to the no-shave group, confirming the external generalizability of CSM’s efficacy [21]. However, cavity shaving does not uniformly reduce re-excision rates. A meta-analysis found no significant reduction in positive margins or secondary surgeries compared to standard excision, except in patients with larger breast volumes [22]. Moo et al. reported that reductions in positive margins were more pronounced in extensive intraductal components or larger tumours, but for T1 invasive cancers without EIC, the benefit was less clear. Additionally, for some surgeons, the reduction in margins may come at the expense of greater tissue excision, which can negatively affect cosmesis and patient satisfaction [23].

#### 3.1.3. Specimen Radiography

Intraoperative specimen imaging methods such as conventional specimen radiography (SR) and intraoperative ultrasonography (IOUSG) provide timely information on whether re-excision of a CSM is indicated during routine BCaS. SR is used to evaluate tissue samples immediately after a biopsy or surgical excision. Traditional SR utilises SR equipment or mammography to image the sectioned tissue with X-rays. SR is performed on a nonpalpable lesion to exploit the X-ray projection of the imaged tissue and produce contrast based on beam attenuation through the tissue [4]. Funk et al. studied 2820 margins each with two planes and assessed the relation between lesion presence with its respective margins. A total of 77.3% of the samples were correctly classified as negative, with a specificity of 86.8% and 37% reduction in secondary operation [24]. Similarly, when Ciccarelli et al. employed SR in the assessment of the status of resection margins in early-stage breast lesions, there was reliability in margin identification (74% positive predictive value (PPV)) and a reduced rate of reintervention from 31% to 20% [25]. The application of SR has opened doors to collaboration with other diagnostic methods. For example, SR was used in association with MRI-guided needle localization of breast tissue. From the eleven out of twelve cases, nine (82%) out of eleven cases with sliced-SRs showed an abnormality resembling that of dynamic contrast-enhanced breast MRI. The pathologist was able to accurately identify the lesion in each of the five malignant cases thanks to sliced-specimen radiographs that displayed the lesion [26]. In a cohort of 170 patients, the accuracy of intraoperative SR using a two-dimensional X-ray device was assessed for margin evaluation. Margins could be evaluated in 91.2% of cases, and relying solely on this technique would have yielded a positive margin rate of 6.5%, achieving negative margins in 93.5% of cases while saving operative time and potentially reducing costs [27]. Despite its diagnostic utility, SR demonstrates limitations. Its sensitivity for identifying positive or close margins is low, with only 36.8% of infiltrated margins correctly identified and a 7% miss rate, despite a high specificity of 86.8%. False positives accounted for 11.7% of margins, potentially leading to overtreatment [24]. For DCIS and microscopic spread in non-calcified or multifocal areas, sensitivity and specificity drop further (72.3% and 52.3%, respectively), with lesion size remaining a dominant predictor of positive margins. Consequently, SR may underestimate residual disease in DCIS despite radiological clearance [28]. Although SR offers important intraoperative guidance, margin clearance is ultimately determined by histopathologic evaluation, which remains essential for identifying subclinical disease beyond the resolution of radiologic assessment.

#### 3.1.4. Intraoperative Ultrasonography

In a prospective observational study of IOUSG, 164 women with palpable breast cancer underwent lumpectomies guided by palpation and ultrasonography. The tumour features and demographics of the patients in both groups were comparable. Despite similar tumour sizes, ultrasound-guided surgery required less removal of healthy tissue and had a lower re-excision rate (6% vs. 17%). The final pathological margins and the margins assessed by intraoperative ultrasound (US) showed a high association. In contrast to conventional palpation-guided excision, the study found that IOUSG is a precise yet efficient way to achieve clear margins while conserving more healthy tissue [29]. Another study examined efficiency, tissue preservation, re-excision rates, and margin status using IOUSG-guided breast-conserving surgery for both palpable and nonpalpable breast tumours. The sensitivity of US localization reached 100% with 95.4% of cases being identified correctly. A total of 91% of patients had negative margins at the initial procedure. with only 2.4% being the definitive positive margin rate. IOUSG-guided breast-conserving surgery is an effective method for obtaining clear margins while minimising unnecessary tissue removal thus lowering the likelihood of repeat procedures [30]. The impact of IOUSG on resection volume during breast-conserving surgery for both palpable and non-palpable breast tumours was studied in Pan et al.’s meta-analysis. Results highlighted precise tumour localization, with excellent resection volumes and better negative margin rates. By reducing the unnecessary removal of healthy breast tissue when compared to conventional methods like palpation-guided surgery and guide wire localization, IOUSG highlighted its ability to be a successful conventional tool for keeping cosmetic results while ensuring oncological safety [31]. The COBALT randomised trial involving 134 patients demonstrated that ultrasound-guided breast-conserving surgery achieved superior cosmetic outcomes and higher patient satisfaction compared with palpation-guided surgery. Notably, 20% of USS outcomes were rated as excellent versus 14% in the PGS group, with fewer poor outcomes (6% vs. 13%) and a significantly lower likelihood of worse cosmetic results in resections < 40 cc [32]. In a retrospective cohort of 250 patients, the use of intraoperative US for margin evaluation significantly increased R0 resection rates, achieving clear margins in 96.4% compared with 82.5% in the control group, highlighting its value in optimising oncological outcomes [33]. A separate prospective study involving 45 patients showed a strong correlation between intraoperative US margin measurements and pathological margins (r = 0.4674, *p* < 0.0008), with margins ≥ 0.5 cm achieving adequate pathology margins in 95% of cases. The authors recommended re-excision of margins < 0.5 cm intraoperatively to minimise reoperation rates, noting reduced accuracy in multifocal tumours [34]. Limitations of IOUSG relate to operator dependency. Technical proficiency in both sonographic analysis and real-time spatial orientation is required, and variability in expertise may limit consistent outcomes [35]. The steep learning curve can deter uptake, particularly in centres without structured IOUS training. Its reliability is also reduced post-NACT, especially in non-palpable tumours, where tissue changes and lack of a palpable mass make localization more difficult. Limited evidence in this setting further restricts its standardisation [36]. A summary of these studies can be reviewed in Table 1. 

### 3.2. Emerging and Device-Based Techniques

#### 3.2.1. Radiofrequency Spectroscopy: MarginProbe Device

To assess the local electrical characteristics of lumpectomy margins in the radiofrequency spectrum, MarginProbe was created. The membrane potential, nuclear morphology, cellular connection, and vascularity of living tissues all influence these electrical characteristics. As a result, it would be possible to distinguish between malignant and healthy areas. By comparing data to pathologic outcomes, the threshold between positive and negative margins has already been established. Surgeons could therefore employ this technique during standard procedures if appropriate [4]. Prospective clinical trials on real-time intraoperative evaluation of lumpectomy margins in 596 breast cancer patients were carried out by Schnabel et al. Patients were randomly assigned to either the device or control arms following the removal of the margins during surgery. MarginProbe was utilised in the device arm to guide further direct excision of positive margins and analyse the primary lumpectomy material. As is currently the norm in most hospitals in America and Europe, surgeons in the control group assessed the cancerous areas that needed to be removed without the use of any instruments. The device and control arms had false-negative rates of 24.8% and 66.1%, and false-positive rates of 53.6% and 16.6%, respectively. According to this intraoperative study, the device arm had 62% of the primary positive specimens, while the control arm had 22% (*p* < 0.001). Consequently, compared to 25.8% in the control group, 19.8% of patients in the device group had a re-excision procedure. The authors point out that the MarginProbe device’s adjunctive usage during BCaS might aid in intraoperative malignancy assessment, hence lowering the necessity for re-excision [37]. In Blohmer et al.’s study, MarginProbe significantly reduced re-excision rates after breast-conserving surgery, with an overall decrease of 14.6%. In patients with DCIS, re-excisions dropped from 61.7% to 23.1%, and in those with invasive lobular carcinoma, from 37.0% to 19.0%. Consistency in utility was observed regardless of tumour morphology, grading, size, breast density, age, BMI, or marker-wire use. It was also able to detect positive margins across different tumour types and resulted in fewer additional surgeries following BCaS [38]. Whilst BCaS aims to eradicate all tumours tissue while simultaneously preserving healthy tissue, 30% of patients require re-excision to achieve negative margins. A total of 46 patients were involved in a prospective randomised trial which evaluated the MarginProbe device as an adjunctive tool in breast cancer therapy. The device group had a significantly lower re-excision rate (4%) compared to the control group (35%, *p* < 0.05) and outperformed previously reported multicentre trial results (19.8%). Despite a higher proportion of DCIS cases in the device arm, surgeons took thicker shavings when MarginProbe indicated positive margins, improving margin clearance and reducing the need for additional surgery [39]. However, several limitations have been noted. In Schnabel et al.’s trial, a discrepancy was observed between positive margin rates (31%) and re-excision rates (20%), potentially influenced by anatomical constraints such as skin or fascial margins and the short two-month follow-up period. The high sensitivity of the device was also associated with reduced specificity, leading to an increase in false-positive results and unnecessary resections [37]. LeeVan et al. reported a high negative predictive value (NPV) (94%) but only moderate sensitivity (67%) and specificity (60%), with a PPV of 16%. With a 2% absolute reduction in re-excision, the added benefit of MarginProbe in settings with already low baseline re-excision rates may therefore be limited [40].

#### 3.2.2. Flouresence Based Margin Assessment

Through enhancing the intraoperative visualisation of desired tissues and structures, fluorescence-guided surgery (FGS) coordinates fluorescent compounds to improve surgical outcomes. During an operation, the surgeon can visualise and differentiate tissues or structures by marking them with the dye [41]. A prospective research involving 35 patients assessing intraoperative margin assessment using indocyanine green (ICG) fluorescence imaging (FI) revealed that 43% of cases had hyperfluorescent signals, whereas 57% had no residual fluorescence. Patients with involved margins showed higher median signal-to-background ratios (SBR) than those with clean margins (1.8 vs. 1.25), and positive margins were found in 14.7% of specimens. ICG-FI imaging sustained an accuracy of 71%, specificity of 60%, and a noteworthy NPV of 100% suggesting that the absence of residual fluorescence reliably predicts clear margins, potentially reducing the need for further intraoperative interventions [42]. Novel approaches like Near-infrared FI can correctly identify tumour fluorescence in all patients. Wang et al. support this investigation, which was able to identify tumour tissue displaying noticeably more intensity than peritumoral and normal tissue (*p* < 0.05). It demonstrated high sensitivity and specificity for detecting positive margins alongside its utilisation intraoperatively as a real-time tool to guide precise excision during lumpectomy, which are supported by the observation of atypical fluorescence in 11.6% of cases, and which is suggestive of residual disease or other benign abnormalities [43]. Shipp et al. created an innovative multimodal imaging method they named multimodal spectral histopathology, which combines tissue autofluorescence (excitation at 405 nm, detection at 450–520 nm) with Raman. Even when analysing large tissue surfaces up to 4 cm × 6.5 cm, they achieved high spatial and spectrum information in about 12 to 24 min by optimising the sampling and data processing algorithms to employ autofluorescence images to drive Raman measurements. Although not conducted under real-time intraoperative evaluation settings, analysis of 121 surgical margin specimens from 107 patients demonstrated an 82% specificity and 95% sensitivity in differentiating invasive ductal carcinoma (IDC) and DCIS from NBG. They stated that it was possible to analyse cancer lesions smaller than 1 mm^2^ [44]. The neutral cholesteryl ester hydrolase 1 (KIAA1363), which is overexpressed in a variety of invasive breast tumours, can be effectively triggered by Fan et al.’s near-infrared fluorescent probe. Through an effective photoinduced electron transfer (PET) process in aqueous buffer solution, the AX11890 compound—the ligand of KIAA1363—is coupled with Nile blue (NB) to extinguish its fluorescence. However, AX11890 separates from the NB dye by a specific distance and regains near-infrared fluorescence (“switch-on”) when the “silent” probe contacts with KIAA1363. Among the biomolecules examined, this probe was found to be quite selective for KIAA1363. Fast recovery of fluorescence was observed, with a detection limit of 0.58 µg mL^−1^ (3δ/k). Within five minutes, the probe was utilised to stain human breast cancer tissue specifically. With excitation at 635 nm, red fluorescent signals could be obtained at depths ranging from 0 to 980 µm. This probe reduces background signals in human tissue samples and has good near-infrared fluorophore qualities for evaluating tumours inside thick tissue samples [45]. The simultaneous fluorescence-based imaging and spectroscopy was accomplished for the classification of IDC tissues and breast cancer margin detection through multimodal devices, which achieved a specificity of 75%, an accuracy of 93%, and a sensitivity of 92.8%. The red shift and maximum fluorescence intensity indicates *p* < 0.01 bolstering its potential as a fluorescence-based intraoperative diagnostic device. The portable, cost-effective, non-invasive, and user-friendly nature of these device all provide a compelling reason for their use in clinical practice [46]. Breast cancer is associated with immunoregulators which contribute to its tumorigenesis. Wilson et al. aimed to utilise antibody-dye contrast agent, B7-H3-ICG to target tumorous breast tissue. B7-H3 expression was higher in tissue with DCIS (46.0 ± 4.8 a.u.) or invasive cancer (91.7 ± 21.4 a.u.) than in normal and hyperplastic tissues (1.3 ± 0.8 a.u.). Tissue pieces classified as normal or hyperplastic had lower average sPA (3.17 ± 0.48 a.u.) and fluorescence signal during image-guided surgical resection than DCIS and invasive carcinoma tissue, which had average sPA (23.98 ± 4.88 a.u.) and fluorescence signals with AUCs of 0.93 and 0.71, respectively. The data concluded that the B7-H3-ICG agent in conjunction with sPA and fluorescent molecular imaging can evaluate the disease status of tissues intraoperatively with excellent resolution, sensitivity, and specificity [47]. Despite encouraging results, several drawbacks exist. Considerable heterogeneity in FI systems across studies, seven different FI systems were used clinically, with some studies employing multiple systems for small cohorts complicates cross-study comparisons [42,48]. Many intraoperative handheld cameras are not optimised for assessing breast cavities, and there is currently no standardised optical imaging platform for this application [49]. Quantitative fluorescence intensity measurement remains challenging, hindering reproducibility [42]. Higher false-positive rates have also been observed in non-specialist centres, emphasising the need for structured training and standardised protocols [50]. Furthermore, limitations of ICG itself—including limited photostability, moderate quantum yield, strong plasma protein binding, undesired aggregation in aqueous solution, and lack of tumour-specific targeting—restrict its clinical potential [51].

#### 3.2.3. Mass Spectrometry and Related Devices

Without the need for molecular labelling, mass spectrometry (MS), a mature analytical technology, can be used to assess a broad spectrum of endogenous compounds in a variety of biological matrices. Despite the wide variety of MS platforms, they are all able to distinguish between molecules according to their mass-to-charge ratios (m/z) [52]. Using MS, Chagovets et al. analysed small tissue samples with accurate identification features to classify cancerous from physiological tissue, achieving a sensitivity and specificity of 100% compared to conventional histology. With a classification time of around 5 min, detailed lipid profiles utilising molecular peaks yielded the best results indicating that tissue spray MS offered a fast and precise tool for intraoperative margin assessment, with potential to reduce repeat surgeries and improve patient outcomes [53]. Direct, quick (around 15 s) molecular analysis of human tissues is possible using the MasSpec Pen (MSPen), a portable, biocompatible instrument that is combined with a mass spectrometer. The method delicately removes endogenous metabolites and lipids from tissues without causing tissue harm by utilising a droplet of solvent. To provide molecular data indicative of tissue type and disease status, the droplet is subsequently moved to and examined by a high-performance mass spectrometer. With the use of this technique, surgeons may identify tissues and make better surgical decisions by non-destructively evaluating the metabolic makeup of tissues in vivo before resection and by examining the removed specimen ex vivo. A system based on MS was assessed for intraoperative margin assessment in surgery for breast cancer. Classification models achieved great accuracy: 95.6% (training), 95.5% (validation), and 90.6% (testing) in an analysis of 143 banked tissue samples (79 healthy, 64 IDC). A total of 273 intraoperative tissue analyses were carried out during 25 breast surgeries, and 147 spectra from 22 of those instances were statistically classified. There was a 95.9% concordance between these intraoperative findings and the ultimate postoperative pathology. These results imply that this real-time MS method offers quick and extremely accurate tissue characterisation and could be a useful intraoperative tool to increase surgical accuracy and decrease re-excisions during breast-conserving operations [54]. Zhang et al. used ex vivo molecular analysis to demonstrate the reliability of the MSPen when separating healthy and malignant tissues across a variety of organs such as the breast. The spectra demonstrated a unique molecular profile made up of proteins, lipids, and metabolites that had an overall accuracy of 96.3%, a sensitivity of 96.4%, and a specificity of 96.2%, highlighting its efficient approach in histologically heterogeneous margin regions. Speed and precision are defining features of the MSPen with promising margin assessment coming from in vivo testing in animal models, which also validated the device’s safety and diagnostic capabilities during surgery [55]. The ambient ionisation method known as Intelligent Knife (iKnife) or Rapid Evaporative Ionisation Mass Spectrometry (REIMS) makes use of the aerosol by-product of electrosurgical instruments. Heat dissipates inside the tissue during the electrosurgical cutting or coagulation process, causing cellular explosion and the release of cellular content into the gas phase. Rapid mass spectrometric chemical analysis is made possible by aspirating the aerosol, and computer algorithms can identify the chemical variations across different tissue types. After electrosurgical activation, the technology can recognise tissue properties in a matter of seconds [56]. A paper studying the utility of iKnife, showed excellent accuracy with the device being able to effectively identify tumour from physiological tissue with 93.4% sensitivity and 94.9% specificity. Further analysis of 260 fresh and frozen specimens revealed a sensitivity of 90.9% and a specificity of 98.8%. Real-time intraoperative feedback was made under two seconds per sample, through its rapid analytical power highlighting the device’s promise for quick and precise intraoperative margin evaluation during breast-conserving surgery [57]. Additionally, Balog et al. showed that the REIMS-based iKnife technology can reliably differentiate between histological and histopathological tumour types based on their lipidomic profiles from more than 2900 tissue samples, as well as accurately distinguish between malignant and non-malignant breast tissues. The technology provided quick and precise diagnostic information during breast surgery coupled with extra information about tumour subtype and biochemistry. In 81 cases of breast cancer resections, intraoperative tissue identification matched the postoperative histology diagnosis in 100% of cases. Its potential to enhance intraoperative margin assessment and direct surgical decision-making is supported by these findings [56]. Certain limitations must be acknowledged. Garza et al. reported false positives in six cases where healthy tissue was misclassified as IDC, likely due to user error, device variability, and molecular differences between fresh and frozen tissue [54]. Intraoperative contamination resulted in signal loss in 16% of analyses, and the MSPen’s limited 5.73 mm^2^ sampling area may have restricted margin coverage, requiring multiple passes. High costs and the need for a mass spectrometer in every operating room also raise questions about scalability [53]. The destructive nature of REIMS prevents histological confirmation, while the iKnife’s 4 mm blade width may dilute tumour cellularity, causing false positives [58]. Its classification of benign lesions remains uncertain, with lower specificity in fibroadenomas despite high sensitivity [57].

#### 3.2.4. Optical Coherence Tomography

The optical form of ultrasonic imaging, known as optical coherence tomography (OCT), uses light waves rather than sound waves to image a living BCaS specimen in its whole. A high-resolution, real-time, multidimensional image of a cancer tissue sample up to 2 mm below the tissue surface can be obtained by applying near-infrared light. The entire live specimen is penetrated by the OCT light, which is then dispersed back to the detector. The scattering properties of cancer tissue are higher than those of fibrous and fatty tissue of normal breast regions because cancer usually has a higher nuclear-to-cytoplasm ratio, higher cellular density, and higher nuclear density. Images of tumours are taken down to 200–1000 µm. OCT imaging is therefore used to distinguish between cancerous and healthy gland tissue [59]. A study assessing the use cross-polarisation OCT was implemented to identify and separate tumorous from non-tumorous tissue in BCaS. Attenuation coefficients were analyses and to assist researchers in forming colour-coded maps to visualise mammary tissue. With the diagnostic accuracy reaching up to 99% and a sensitivity reaching 99%, tumour cell and stroma identification proved to be both fast and dependable during breast-conserving surgery [60]. To ensure that the depth of the lens’s field (1.47 mm) matches the depth of OCT penetration in the entire BCaS specimen, Nguyen et al. evaluated the surgical margins of lumpectomy specimens using their custom-made, needle-based OCT probe. Additionally, their equipment has a high-resolution scanner that produces better images. The sensitivity was 100% and the specificity was 82% when OCT-based breast cancer surgical margin data and pathologic technique data were compared (9 true positives, 9 true negatives, 2 false positives, and 0 false negatives). These findings demonstrate the possibility of OCT imaging for breast cancer marginal assessment [59]. In a similar study, after a training period of around 3.4 h, readers from a multi-disciplinary team (radiologists, pathologists, and surgeons) distinguished suspicious areas with an average sensitivity of 80%, specificity of 87%, and overall accuracy of 87%. These findings suggest that OCT imaging can be effectively interpreted by various specialists with minimal training, supporting its feasibility as a real-time tool to aid intraoperative margin evaluation in breast-conserving surgery [61]. Wide-field-OCT was investigated on 185 tissue samples as a tool to evaluate tissue margins which included lumpectomy specimens and margin shaves. By achieving an accuracy of 96.2%, these findings demonstrate a strong correlation between WF-OCT imaging and pathological margin status. Most discordant cases involved close margins (<2 mm), mostly seen in DCIS. Initial data provided compelling support regarding its feasibility as a real-time intraoperative tool suggesting that Wide Field-OCT could help reduce re-excision rates by improving margin assessment accuracy [62]. OCT is constrained by its shallow penetration depth (~1.5 mm) and the relatively small scanning area, which can limit its applicability to larger specimens [60]. Optical signal variations due to coagulation and haemorrhage can complicate in vivo interpretation, and tissue shrinkage during fixation hampers precise histological co-registration. In some cases, high-density tumour borders and adipose tissue exhibit similar attenuation values, making delineation challenging. Additionally, point-by-point scanning methods with slow acquisition rates are impractical for imaging entire specimen surfaces while maintaining high resolution, highlighting the need for faster, wide-field techniques [63].

#### 3.2.5. AI Advances in Margin Assessment

**Figure 2:**
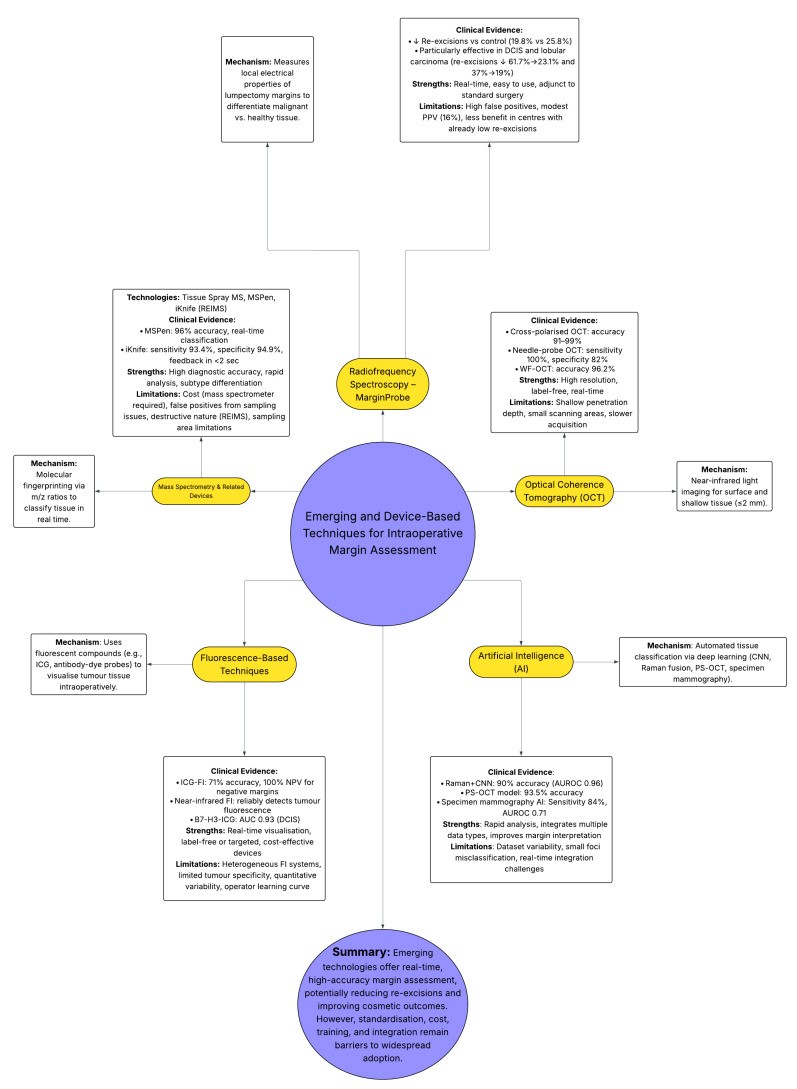
Overview of emerging and device-based intraoperative margin assessment techniques, including radiofrequency spectroscopy, optical coherence tomography, fluorescence imaging, mass spectrometry-based tools, and artificial intelligence. Each method’s mechanism, key evidence, strengths, and limitations are summarised, highlighting their potential to improve real-time margin evaluation and reduce re-excision rates.

Using sophisticated non-linear mathematical simulations and basic components that resemble human neurones, artificial intelligence (AI) aims to build autonomous systems that can carry out tasks that have historically been completed by people. Beginning with the perception, understanding, and execution of cognitive processes—such as intellect, creativity, language comprehension, memory, pattern recognition, vision, reasoning, and connecting information—this discipline investigates how the human mind works. AI seeks to mimic these skills to do a variety of jobs, ranging from simple ones like object identification to more intricate ones like predicting [64]. AI techniques include using statistical models, forecasting future events, and learning from current data without bias. This method improves decision-making by increasing its efficiency and level of knowledge. A study fused Raman spectroscopy with a convolutional neural network (CNN) which effectively distinguished cancerous and normal breast tissue with 90% accuracy, 88.8% sensitivity, and 90.8% specificity (AUROC 0.96). By creating a clear visualisation of tissue margins, which were reinforced with traditional histology, key molecular features could be mapped using specific Raman peaks, producing high-resolution, colour-coded tissue images that highlight differences between adipose, connective, and tumour tissues. The findings support the potential of Raman spectroscopy as a swift, label-free, and accurate imaging method for both research and real-time intraoperative margin assessment during breast-conserving surgery [65]. Santilli et al. aimed to bolster the performance of the iKnife by building a self-supervised learning model from limited, weakly labelled data. The general properties of iKnife data from a more accessible cancer kind can then be contextualised by the model. Second, a cancer classification task using breast data can be applied to the trained model. Learnt weights can be transferred from models of one tissue type to another thanks to this domain adaptation. The final model achieved 92% accuracy, 88% sensitivity, and 92% specificity—significantly outperforming a baseline approach reinforcing its feasibility of enhancing iKnife performance with limited breast data thus supporting its potential for real-time intraoperative margin assessment in breast-conserving surgery [66]. Deep Ultraviolet (DUV) fluorescence scanning microscopy has recently been expanded through the integration of an ensemble deep-learning approach which automates margin evaluation during BCaS. By examining whole surface images at a patch level, the technique successfully localised cancerous vs. benign tissue with 95% accuracy and 100% sensitivity in detecting malignant areas. According to these results, the suggested method performs better on DUV pictures than conventional deep learning techniques and may improve intraoperative margin evaluation, guaranteeing total cancer removal while protecting good tissue and, eventually, lowering the need for reoperation [67]. Zhu et al. developed an automated model to classify malignant tumour, fibro-adipose, and stromal tissue in breast specimens using polarisation-sensitive optical coherence tomography (PS-OCT). The model was derived from standard OCT intensity and four polarisation-based metrics, which enabled it to obtain an accuracy of 93.5%, outperforming a model using intensity-only features (82.9% accuracy). This underscores the value of polarisation information in distinguishing breast tissue types with additional real-time utility to aid surgeons in accurately differentiating tissue during BCaS directly in the operating theatre, inevitably improving margin assessment and reducing reoperation rates [68]. SR has also been subject to AI enhancements, with researchers pretraining models on radiographic images to predict pathological margin status from specimen mammography images in breast-conserving surgery. With 821 mammograms paired with known margin outcomes, Chen et al.’s best-performing model (InceptionV3) yielded a sensitivity of 84%, specificity of 42%, and an AUROC of 0.71. With performances edging towards invasive cancer and lower breast density tissue, the AI can outperform current interpretive methods and, with further refinement, may assist surgeons intraoperatively in improving margin assessment and reducing reoperation rates [69]. Current AI applications face several challenges. Variability in specimen size, inconsistent orientation, and limited, homogeneous training datasets reduce model generalizability [70]. Small malignant foci within larger benign areas may be misclassified, particularly when patch labels cover only part of the tissue [67]. Additionally, many models fail to incorporate geographic and demographic diversity, potentially limiting broader applicability. Real-time feedback integration during surgery remains technically demanding, hindering intraoperative clinical translation [68]. Future applications of AI may focus on integrating preoperative radiological data—particularly from MRI and contrast-enhanced mammography—with intraoperative specimen analysis to better predict margin status. By identifying high-risk features associated with positive margins, such multimodal AI models could support tailored surgical strategies and improve margin-negative resection rates. Table 2 and Figure 2 highlight the emerging techniques and device-based margin assessment techniques. 

## 4. Discussion

To impact reoperation rates, patient outcomes, and aesthetic preservation, margin assessment must be improved in BCaS. Our review aims to address both conventional and emerging technologies in this field and highlight both their clinical utility and inherent challenges.

With FSA and CSM already proving to be pivotal, both these tools are highly utilised for their reliability within the operating theatre. With FSA demonstrating high diagnostic accuracy, with sensitivity reaching 96% and specifically over 84%, it enables immediate intraoperative re-excision and significantly reduces reoperation rates. Studies like Agarwal et al. and Nowikiewicz et al. reinforce their value in achieving oncologically complete surgeries while minimising cost and patient distress. Despite this, with technical challenges present, it limits the scope of their impact when issues such as prolonged operative times, technical artefacts, and interpretive challenges result in stagnation in their performance in the operating theatre. Similarly, CSMs have shown a consistent reduction in positive margin rates and re-excisions, without compromising cosmetic outcomes. As medicine advances, approaches to smaller and localised lesions become increasingly accessible. This issue cannot be directly addressed by existing methods where benefit appears most pronounced in patients with EICs or larger tumour volumes [9,17,18,19,20,21,22].

Intraoperative imaging techniques, including SR and IOUSG, offer real-time guidance and tissue conservation. IOUSG has shown promise in minimising resection volumes and improving cosmetic results while preserving oncologic safety. However, its utility is highly operator-dependent, and its effectiveness may be reduced following neoadjuvant chemotherapy due to altered tumour morphology. Similarly, when assessing SR, the reduction in reoperation rates appears its driving argument of utility; however, when diving into its sensitivity for microscopic and diffuse pattern detection such as DCIS, it performs inconsistently. False positives can further result in overtreatment and poor cosmesis [14,17,23,24,25,26,27,28,29,30,31,32,33,34,35,36].

It is important to distinguish margin standards between invasive carcinoma and DCIS. Current consensus guidelines define a negative margin for invasive breast cancer as “no ink on tumour,” whereas for DCIS, a margin of ≥2 mm is recommended to minimise the risk of local recurrence. These criteria were applied when analysing the studies included to ensure consistency.

Emerging technologies aim to address a vast field of hindrances which impact conventional approaches ranging from molecular-level approaches, high-resolution, and sometimes label-free diagnostic capabilities. Radiofrequency-based tools like MarginProbe have consistently reduced re-excision rates, especially in DCIS and invasive lobular carcinoma, despite high false-positive rates. FGS, including ICG and B7-H3-based agents, introduces intraoperative visual differentiation of malignant and benign tissues, with strong NPVs and favourable SBRs. Within FI, variability exists. This coupled with a lack of standardised protocols hinder widespread adoption [37,38,39,40,41,42,43,44,45,46,63,64,65,66,67].

MS tools such as the MSPen and iKnife represent innovative innovations that combine rapid molecular analysis with intraoperative feedback. Studies have shown high concordance with final histology and significant diagnostic precision. However, high costs, technical complexity, and destructive sampling (as seen in REIMS-based systems) limit routine clinical implementation. Furthermore, contamination, user variability, and a need for larger sampling areas must be addressed to improve reliability [47,48,49,50,51,52,65]. The wide-field or polarisation-sensitive forms of OCT provide excellent accuracy rates, reaching up to 95% in some studies. With its near-histological imaging with rapid feedback, it places the tool as an ideal choice for intraoperative use. However, challenges such as shallow penetration depth, interference from tissue artefacts, and difficulties with one-to-one histological mapping reduce its overall practicality. Given the continuous advancements in the field of elastography-based OCT or AI-enhanced imaging, these technical obstacles could be mitigated [53,54,55,56,59].

AI has revolutionised medicine throughout various diagnostic modalities. Within margin assessment, AI has bolstered CNNs analysing Raman spectroscopy and domain-adapted models enhancing devices like the iKnife enhancing margin prediction, sensitivity, and specificity. Despite this, the generalisability of these models is down to limiting training databases, varying specimen orientation and a lack of diversity amongst databanks. Further challenges include interpreting mixed tissue types and overfitting which can prolong its integration within modern medicine [57,58,59,60,61,62,70].

Irrespective of technological advancements, achieving perfect accuracy is a demanding task which cannot be obtained without rigorous testing and diverse datasets. Each approach bears limitations related to cost, operator dependency, processing time, or interpretability. Therefore, a multimodal strategy—integrating imaging, molecular analysis, and AI interpretation—may offer the most robust and flexible intraoperative assessment framework. Ensuring that training protocols are standardised is essential to refining these modalities. Alongside this, broader multicentre trials, and cost–benefit analysis both benefit the applicability of emerging tools in clinical practice.

## 5. Conclusions

Surgical success can be defined by a multitude of factors, but margin assessment maintains a critical role in the determination of surgical outcome, patient satisfaction and oncological safety. Conventional techniques like FSA, CSM, SR, and IOUSG have all solidified their utility in the operating theatre by reducing re-excision rates and preserving cosmetic outcomes. Despite this, issues ranging from limited sensitivities to prolonged operation times emphasise the margin of improvement which must be filled by advancing medicine.

Devices including MarginProbe, fluorescence-guided surgery, mass spectrometry-based tools (e.g., MSPen, iKnife), OCT, and AI-augmented imaging systems have achieved excellent results regarding real-time feedback, diagnostic accuracy and tissue preservation. By addressing problems with more conventional methods, it paves a path for a more streamlined approach to patient care. To achieve this, variability in data interpretation, high application cost and extensive training times hinder its general use.

Investment in standardised protocols, surgeon training, and large-scale validation studies will be essential to ensure consistent clinical impact. With advances in modern medicine, synergy between imaging, spectroscopy, and AI may provide a pivotal opportunity for enhanced patient care and surgical outcomes, which inevitably improves the quality of life for patients with breast cancer.

## Figures and Tables

**Table 1:** Summary of pivotal studies evaluating conventional intraoperative margin assessment techniques in breast-conserving surgery, including frozen section analysis, cavity shave margins, specimen radiography, and intraoperative ultrasonography. The table outlines study design, sample size, principal findings, and overall conclusions regarding their diagnostic performance and clinical impact.

Study (Author, Year)	Technique	Design and Sample	Key Results	Conclusion
Nowikiewicz et al., 2019 [10]	Frozen Section	Retrospective cohort, 1102 patients	Intraoperative frozen section was used in 45.8% of cases (505/1102) False-negative margin rate: 14.3% (with FSA) vs. 16.9% (without) Conversion to mastectomy: 5.9% (with FSA) vs. 9.7% (without) Surgery duration: 48.9 min (with FSA) vs. 42.9 min (without)	The use of frozen section during breast-conserving surgery reduced reoperations for residual tumour but significantly increased the duration of surgery.
Agarwal et al., 2023 [11]	Frozen Section	Cohort, 328 patients	18% had margins detected by frozen section histology, enabling immediate re-excision and avoiding reoperation. 2.4% false negatives on frozen section histology. Without frozen section histology, more reoperation needed (*p* < 0.001).	Use of frozen section histology enables one-step oncologically complete breast-conserving surgery in most patients, significantly reduces costs by avoiding reoperations, and prevents patient anxiety and delays in adjuvant treatment.
Chagpar et al., 2015 [19]	Cavity Shave	RCT, 235 patients	Positive margins: 19% vs. 34% Re-excisions: 10% vs. 21%	CSM halves margin positivity and reoperation rates.
Dupont et al., 2021 [21]	Cavity Shave	Multicentre RCT, 396 patients	Post-randomization positive margins: Shave 9.7% vs. No-shave 36.0% (*p* < 0.001) Re-excision/mastectomy rates: Shave 8.7% vs. No-shave 23.5% (*p* < 0.001)	Confirms CSM effectiveness across multiple centres.
Funk et al., 2020 [24]	Specimen Radiography	Multicentre, 470 cases	89.0% margins were negative: 11.0% positive Sensitivity: 36.8%; Specificity: 86.8% PPV: 25.6%; NPV: 91.8% Positive margins reduced from 11.0% to 7.6% via targeted re-resections Secondary procedures reduced by 37.0% (from 34.5% to 21.7%)	SR helps identify positive margins and reduces the need for secondary surgeries through targeted re-excisions.
Ihrai et al., 2014 [27]	Specimen Radiography	Prospective,170 patients	Margin status was evaluable in 91.2% of cases using IOSR. If based solely on IOSR, positive margin rate would have been 6.5%. Negative margins are potentially achieved in 93.5% of cases.	SR effective for intraop margin confirmation with an additional time saving and economic impact.
Karanlik et al., 2015 [29]	IOUSG	Prospective cohort, 164 patients	US-guided lumpectomy had a lower re-excision rate (6%) than palpation-guided surgery (17%). Closest margins by US strongly correlated with pathology margins (r = 0.76).	IOUS reduces reoperation and aligns well with pathology.
Haloua et al., 2016 [32]	IOUSG	RCT, 134 patients	IOUSG showed better cosmetic results: 20% rated excellent vs. 14% in palpation-guided surgery. IOUSG had fewer poor outcomes (6%) compared to palpation guided surgery (13%).	IOUS improves margin control and reduces tissue removal.
Eichler et al., 2012 [33]	IOUSG	Retrospective, 250 patients	Among 250 breast-conserving surgeries, 33.6% used intraoperative ultrasound (IOUS). Clean margins (R0) were achieved in 87.2% overall. IOUS group had a higher rate of R0 (96.4%) compared to control (82.5%), a significant difference.	Increase in R0 resection rates when intraoperative ultrasound was used to evaluate surgical margins.
Olsha et al., 2011 [34]	IOUSG (specimen US)	Prospective cohort, 45 patients	31% had intraoperative margin re-excisions, mostly toward closest pathological margin. 7% required a second surgery despite intraoperative re-excision, with no residual cancer found. Ultrasound margins ≥ 0.5 cm predicted pathology margins ≥ 0.2 cm in 95% of cases.	Specimen US reliably predicts final margins and IOUSG helps reduce reoperation rates after breast-conserving surgery.

**Table 2:** Overview of emerging and device-based intraoperative margin assessment techniques, including radiofrequency spectroscopy, optical coherence tomography, fluorescence imaging, mass spectrometry-based tools, and artificial intelligence. Each method’s mechanism, key evidence, strengths, and limitations are summarised, highlighting their potential to improve real-time margin evaluation and reduce re-excision rates.

Study (Author, Year)	Technique	Design and Sample	Key Results	Conclusion
Schnabel et al., 2014 [37]	Radiofrequency Spectroscopy	Randomised Clinical Trial, 596 patients	False-negative rate: 24.8% (device) vs. 66.1% (control). False-positive rate: 53.6% (device) vs. 16.6% (control) Positive margin resection (main specimen): 62% (device) vs. 22% (control) Re-excision rate: 19.8% (device) vs. 25.8% (control); 6% absolute, 23% relative reduction	MarginProbe aids intraoperative margin assessment and reduces reoperation.
Blohmer et al., 2016 [38]	Radiofrequency Spectroscopy	Prospective Clinical Trial, 150 patients	Overall re-excision rate reduction: 14.6% DCIS subgroup: 61.7% → 23.1% Invasive lobular carcinoma subgroup: 37.0% → 19.0%	Device is effective regardless of tumour type, patient factors.
Wang et al., 2022 [43]	Fluorescence based margin assessment	Imaging study, 43 patients	Fluorescence detected: 100% of primary tumours. Average fluorescence intensity (a.u.): ○ Tumour: 219.41 ± 32.81 ○ Peritumour: 143.35 ± 17.37 ○ Normal: 105.77 ± 17.79 SBR: ○ Tumour/peritumour: 1.54 ± 0.20 ○ Tumour/normal: 2.14 ± 0.60	Fluorescence shows promise as a real-time margin detection tool.
Shipp et al., 2018 [44]	Fluorescence based margin assessment	107 patients, 121 specimens	Sensitivity 95%, specificity 82%	This multimodal approach may offer an objective tool for intraoperative margin assessment, helping reduce unnecessary reoperations.
Fan et al., 2017 [45]	Fluorescence based margin assessment	In vivo probe testing	Detection limit 0.58 µg/mL	Selectively lights up invasive tissue in thick samples.
Wilson et al., 2018 [47]	Fluorescence based margin assessment	Tissue analysis	AUCs 0.93 (DCIS) and 0.71 (invasive carcinoma tissue)	High sensitivity and resolution support intraoperative use.
Chagovets et al., 2020 [53]	Mass Spectrometry and related devices	Validation Study, 50 samples	100% sensitivity and specificity	Fast, accurate tool for intraoperative assessment.
Zhang et al., 2017 [55]	Mass Spectrometry and related devices	Animal and human validation	High sensitivity (96.4%), specificity (96.2%), and overall accuracy (96.3%)	Promising for real-time tissue classification.
Balog et al., 2013 [56]	Mass Spectrometry and related devices	302 patients	100% match with histology	Reliable tools for rapid intraoperative diagnosis.
Nguyen et al., 2009 [59]	Optical Coherence Tomography	Lumpectomy margins, Clinical Trial of 37 patients	Sensitivity 100%, specificity 82%	OCT is viable for high-resolution intraoperative guidance.
Gubarkova et al., 2023 [60]	Optical Coherence Tomography	Diagnostic imaging with 68 freshly excised human breast specimens	Att(cross) for tumour vs. fibrous tissue: ○ Accuracy: 91–99% ○ Sensitivity: 96–98% ○ Specificity: 87–99% Att(co) for tumour vs. adipose tissue: ○ Accuracy: 83% ○ Sensitivity: 84% ○ Specificity: 84%	Reliable tumour-stroma differentiation in surgery.
Chen et al., 2023 [69]	AI Advances in margin assessment	821 paired mammograms	Best model (InceptionV3): ○ Sensitivity: 84% ○ Specificity: 42% ○ AUROC: 0.71 Better performance in patients with invasive cancer, less dense breasts, and non-white race	AI may improve margin assessment from specimen imaging.
Santilli et al., 2021 [66]	AI Advances in margin assessment	Transfer learning model with 62 patients	Accuracy 92%, sensitivity 88%	AI boosts iKnife performance in data-limited settings.
Zhu et al., 2021 [68]	AI Advances in margin assessment	Image classification with 720 images from 41 patients	PS-OCT model accuracy: 93.5% Standard OCT (intensity-only) model accuracy: 82.9%	Polarisation information significantly improves breast tissue differentiation

## Data Availability

Not applicable.

## Abbreviations

The following abbreviations are used in this manuscript:

AI	Artificial Intelligence
AUC	Area Under the Curve
AUROC	Area Under the Receiver Operating Characteristic Curve
ALN	Axillary Lymph Nodes
BCaS	Breast Cancer Surgery
CSM	Cavity Shave Margins
FI	Fluorescence Imaging
FSA	Frozen Section Analysis
IDC	Invasive Ductal Carcinoma
MS	Mass Spectrometry
MSPen	MasSpec Pen
NPV	Negative Predictive Value
OCT	Optical Coherence Tomography
PPV	Positive Predictive Value
RCT	Randomized Controlled Trial
R0	Complete (Negative) Resection
SBR	Signal-to-Background Ratio
SR	Specimen Radiography
US	Ultrasound
